# Thunderstorm Asthma Epidemic—A Systematic Review of the General Practice Perspective

**DOI:** 10.3390/ijerph17113796

**Published:** 2020-05-27

**Authors:** Ambereen Farouque, Rae Walker, Bircan Erbas

**Affiliations:** 1 Department of Public Health, School of Psychology and Public Health, La Trobe University, Bundoora, 3086 VIC, Australia; amber.farouque@gmail.com (A.F.); r.walker@latrobe.edu.au (R.W.); 2 Faculty of Public Health, Universitas Airlangga, Surabaya 60115, Indonesia

**Keywords:** thunderstorm asthma, general practice, disaster management

## Abstract

Thunderstorm asthma (TA) epidemics have been recognized globally as a rare phenomenon, producing a rapid surge of acute asthma presentations leading to an increased demand on emergency medical services and healthcare resources. General practitioners (GPs) are well placed in the community to contribute to healthcare during TA epidemics and similar disaster events. The aim of this review was to synthesize current evidence of the experiences of GPs during TA epidemics and similar surge events. A comprehensive systematic search of eleven electronic databases, including ancestry searching for peer-reviewed studies and grey literature published in English was conducted. Quantitative and qualitative study designs were included, and a quality assessment conducted. Of the 125 records identified, 16 were included for synthesis. During TA epidemics and surge events, GPs experience an increased demand for services, yet it is not known if general practice clinics experience resource limitations from this patient surge. While GPs express a willingness to help, few structures are in place to liaise, support and provide information to GPs during surge events. Following most surge/disaster events, no GP data is collected so it is not known how to improve coordination and communication between general practice services and emergency services. GPs have well-functioning adaptive management systems, and resources of space, supplies and staff thus the ability to increase surge capacity of their clinics.

## 1. Introduction

Thunderstorm Asthma (TA) epidemics are a rare but increasingly significant public health challenge globally [1,2,3]. Australia has experienced 10 of the 22 of TA epidemics recorded to date, the most recent one in Melbourne, in 2016. In just 12 h, a single storm placed an unprecedented strain on available healthcare resources, resulting in the death of ten patients and 10,000 asthma related presentations to health services [4]. The challenge faced by emergency response services indicates that it is time to examine the role of all emergency healthcare providers in delivering solutions required to manage surge events of this type. General practitioners GPs are well placed in the community to respond to public health emergencies of this scale yet the general practice sectors’ role in disaster response is poorly defined [5].

Although asthma is primarily managed by GPs, very little is known about TA epidemics and their impact on general practice. Only two association studies have documented the role of GPs [6,7]. During the evening of the 1994 UK thunderstorm event, a general practice deputising service reported an increase in asthma callouts by 1500 from a single afterhours GP service [7]. In another UK based study, authors reported a sixfold increase in asthma attendance to general practice services the day after the thunderstorm [6]. Several studies have investigated the health services response to this phenomenon, cataloguing the strain on health services and resources in a hospital setting (ED, ICU, hospital admissions) [8,9]. However, some insight can be gained about TA from the general practice response to other surge events, disasters and epidemics.

The only other systematic review in this field reports that primary health care (PHC) is an important aspect of disaster response and recovery, disaster risk reduction and community preparedness, yet readily concedes there are a relatively limited number of published works. Current research is “lacking in a multi-disciplinary, multi-sector co-authorship” [10]. Literature is very much descriptive of single events by single authors. There are examples in Canada, the US and the UK of building capacity for community disaster risk reduction using PHC infrastructure. However, only passing comment is made about the challenges of intra-sector communication and collaboration (especially with health departments and public health bodies).

Why are studies regarding the general practice response so limited? That thunderstorm asthma is a newly emerged, poorly understood and rarely occurring phenomenon only partially explains the gap in the research. Historically, the general practice sectors’ role in disaster response is poorly defined and largely unacknowledged [5,11]. Prior to 2000, some authors felt that in an urban setting with well-functioning emergency medical systems and short transport times there may be little, if any, role for the GP [11,12]. In more recent years, however, the vulnerability of communities to mass casualty and surge events has been acknowledged along with recognition of the importance of general practice in the emergency medical response [13].

To be able to conduct a meaningful review, the literature search was broadened to include other types of public health emergency events relevant to general practice, so that an adequate amount of pertinent material could be located. Although a systematic review is the preferred and followed approach, we extended this by incorporating a wide selection of materials from experimental, non-experimental and grey literature simultaneously. This method summarises past empirical and theoretical literature and gives a comprehensive account of a rare phenomenon in an infrequently studied group.

The purpose of this review is to describe and qualitatively synthesize the evidence concerning the experience of GPs and practice nurses (PNs) (who work alongside GPs in PHC setting) during TA epidemics and public health emergencies similar to TA epidemics. Our review will identify the major themes in the current literature, as well as the lessons learned, gaps and future directions for research.

## 2. Materials and Methods

A systematic review was conducted following the development of a protocol by researchers. An electronic literature search applied adapted PRISMA guidelines for reporting systematic reviews of qualitative and quantitative evidence [14]. This review was not registered.

### 2.1. Search Strategy

A systematic search of peer-reviewed literature following PRISMA guidelines was performed from inception up to November 2019. Electronic databases searched included: CINAHL, MEDLINE, PubMed, SCOPUS, Web of Science, Global Health, Science direct, ProQuest Dissertations and Theses Full Text, ProQuest Health and Medicine, Trove and Google Scholar (first 10 pages). Google Scholar can be particularly helpful when searching multidisciplinary topics, where the search journal can reasonably be expected to span multiple subjects and be indexed in multiple data base [15]. All grey literature resulting from the Google Scholar search was also reviewed. All reference lists of the identified reports and articles were ancestry searched for additional papers.

### 2.2. Search Terms Used for the Search Strategy

Combinations of keywords and medical subject headings (MeSH) were used. Articles were shortlisted, if any combination of words appeared anywhere and were in English. Search terms using; general practice (general practitioner) [Australia and UK], family practice (family physician) [USA and Canada], combined with thunderstorm asthma (epidemic), OR public health emergency, public health disaster, disaster, pandemic influenza, respiratory pandemics, bushfire, forest fire, largescale industrial fires, cyclone, hurricane, flooding and heatwaves. The term “emergency” was not used as it resulted in a large number of unrelated topics. Supplementary Materials Figure S1 is an example of a Medline search strategy.

Broadening the search terms by combining thunderstorm asthma (epidemic) AND emergency department, general practice AND health services response provided articles about responses of health services to this unique event type.

### 2.3. Inclusion Criteria and Definitions

Primary study participants: general practice doctors and practice nurses who have dealt with public health/disaster surge events. Emergency department staff who experienced a TA epidemic.

**Figure 1:**
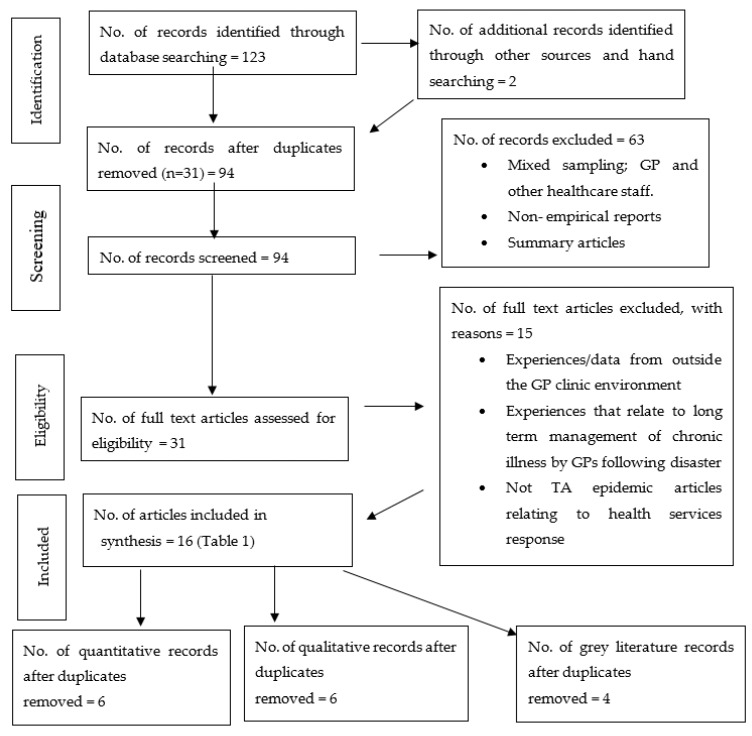
PRISMA flow chart of the study selection process.

Outcome measure or phenomena of interest: experience of staff during a TA event OR a public health emergency OR a public health disaster similar to a thunderstorm asthma event OR any type of public health disaster event where GPs experienced an acute surge in the patient load. Research methodologies: There was no limit on the study design (qualitative, quantitative and mixed method studies). This review also considered the grey literature. Published in the English language only. Our review excluded non-English studies. We accessed English language articles using the guidelines outlined by the Cochrane Handbook for Systematic Reviews of Interventions [16]. Time period included: 2000 up to current for general practice/public health emergency/experience. No time limit was placed on the articles for thunderstorm asthma. Date of review: 1st July 2017 until 1st November 2019. The search strategy aimed to find both published and unpublished papers. Reasons for exclusion are listed in Figure 1.

### 2.4. Selection of Included Articles

The abstracts of all identified papers were reviewed for initial inclusion by authors AF and RW; then full papers were read by AF to determine if all inclusion criteria were met. Articles were shortlisted, if any combination of words appeared anywhere and were in English.

### 2.5. Data Extraction

Data was extracted using a standardized method. Data extracted from each study included: author, year of publication, study design (qualitative, quantitative or grey articles), study population, number of study subjects (where applicable), country where the study was conducted, event type (disaster/patient surge event). Criteria included; rapid onset event, large numbers of patients seeking care from general practice, no/minimal long-term impact of the disaster/surge event, minimal or no deaths faced by general practice services. Criteria were chosen to focus on literature that reflects themes specific to mitigation, preparedness and response challenges that best apply to TA epidemics. Articles that focused on the long-term impact (e.g., mental health care post-disaster) and major casualty/mortality were excluded, as they highlighted themes of less relevance to the topic overall. Major themes were extracted from each article. Each theme relates to what may constitute an experience, attitude, opinion, challenge, role or response, see Supplementary Materials Table S1.

### 2.6. Data Evaluation and Assessment of Quality

The methodological quality of the studies included in this review were formally assessed. However, assessing grey literature sources like policy documents, royal commissions, government inquiry findings with qualitative research was problematic. Grey literature can be variable in its quality and relevance and was therefore assessed using an appraisal tool for public health grey literature: Public Health Ontario guide to appraising grey literature [17] (see Supplementary Materials Table S2). All the grey material reports scored highly upon assessment. A separate flexible appraisal tool was applied to quantitative, qualitative and mixed methods articles: MetaQAT (quality appraisal tool) [18] (see Supplementary Materials Table S3). Nine of the twelve mixed methods studies scored highly on a 2-point scale (high or low). The scale was based on methodological rigor and data relevance, and no report was excluded based on the evaluation. However, the score was applied as a variable in the data analysis stage. Articles of low rigor and relevance contributed less to the analysis process overall [19]. Thus, in the development of the tables of themes, articles of low relevance, reliability, validity and applicability contribute less to the process of developing major themes.

## 3. Results

No review was located on this topic. The comprehensive search resulted in 94 peer-reviewed scientific articles after duplicates were removed, (see Figure 1) Of these, 63 were irrelevant and removed following screening of the titles and abstracts. A substantial number of excluded papers were irrelevant to the review because they were a mixed sampling of general practice doctors/nurses with other healthcare workers where the GP contribution could not be isolated, articles not reporting the actual and direct experience of general practice staff, non-empirical reports or peer reviewed summary articles.

Finally, 31 full text articles were assessed and 15 were again excluded. These excluded articles reported experiences outside the GP environment, management of chronic illness post-disaster or a TA epidemic article not relating specifically to a health service’ response.

In total, 12 mixed methods articles and 4 grey literature documents were included in the review and their characteristics listed in Table 1.

### 3.1. Increased Demand for Services

GP and ambulance data all indicate an increased demand on GP services during a TA epidemic. Two general practice studies that examined the same thunderstorm asthma event in the UK uncovered an increased demand on general practice services during the thunderstorm [7] and the day after [6]. As the 2016 TA epidemic unfolded in Melbourne, time critical referrals from general practitioners to ambulance services increased by 47% (21% to 80%) [20]. A sustained increase in demand for general practice services occurred in Australia during several recent public health emergency events; mine fire, bushfires and pandemic influenza [21,22,23]. After the New Zealand earthquake GPs indicated that the process for identifying hotspots of increased general practice service demand was poorly coordinated [24].

### 3.2. Limited Resources

Limited resources are a clear feature of the health services impact of a TA epidemic as described in Supplementary Materials Table S1. The only study investigating the impact on health services’ resources by a thunderstorm asthma event uncovered an unprecedented demand on emergency department (ED) resources when ten times the expected number of patients attended hospitals seeking treatment in just 30 h [8]. Half of the London hospital EDs surveyed ran out of supplies of equipment and medication. These included standard supplies for asthma including nebulizer face masks, beta agonist nebules, beta agonist inhalers and prednisolone. All EDs called in additional staff, both medical and nursing. Limited space to accommodate respiratory patients also proved challenging during the patient surge [8]. It is likely that GP clinics would experience a similar resource limitation.

Limited resources impacting the general practice response to public health emergencies has not been investigated in the literature to any great extent. Interviews with general practice staff indicate that most GPs feel underprepared for disasters [25,26,27]. Hogg et al. questioned Canadian family physicians (GPs) about respiratory pandemic influenza found only 18% feel adequately prepared with regards to resources. [26]. The majority of Australian GPs in the Tasmanian setting were concerned about limited personnel during respiratory surge events stating “the general practice workforce could easily become overwhelmed” [27], p. 270. GPs also expressed concerns regarding practice preparedness issues especially limited equipment [27], limited health services infrastructure and personnel [28].

### 3.3. Willingness to Help

Willingness of GPs and their staff to provide assistance during a public health emergency or disaster is reported by most studies [24,25,26,27,29]. All enquiries into Australian disasters comment upon this willingness of nurses and doctors in general practice to assist in patient care outside their normal practice parameters [21,22,23]. Australia’s experience with bushfires, respiratory pandemics, industrial fires and thunderstorm asthma shows the willingness of GPs to help patients during such events yet very few authors comment on the factors facilitating or hindering a “willingness to help” (see Supplementary Materials Table S1) [21,22,23].

### 3.4. Enhancing Surge Capacity through Communication and Co-Ordination

The potential for enhancing the surge capacity during disaster events through the participation of general practice clinics is proposed by Hogg et al. [26]. Physician respondents (Canadian GPs) indicated that their clinic resources could significantly enhance the emergency response during respiratory pandemics [26]. Hogg’s participants were happy to be notified of public health emergencies via multiple communication platforms, (see Table 1, reference [2]) Yet, Hogg admits that there are very few systematic structures in place to facilitate general practice involvement in public health emergency management [26]. US family physician (GP) respondents that were surveyed overwhelmingly felt information dissemination between local health departments and GPs during infectious pandemics was inadequate [30]. Multiple grey literature inquiries into disaster events in Australia have noted the importance of co-ordination between emergency health authorities and GPs. Yet most of these reports readily admit that very few structures are in place to liaise, support and provide information to GPs [21,23,28]. More often there is very little mention of the GP’s role during disaster events and usually no data is collected from general practice services.

### 3.5. Techniques to Manage Public Health Emergencies in a General Practice Setting

Pitts et al. noted the need to delay the usual procedures and processes present at clinics to facilitate the care of a large surge in patient numbers [29]. Developing criteria-based treatment including triage and rationing were techniques used by general practice trainees in an evolving scenario approach when presented with an influenza pandemic scenario [29]. Edwards et al. also noted similar methods employed by family physicians who treated hurricane survivors in the U.S. [25].

## 4. Discussion

To our knowledge, this is the first systematic review to present evidence of the experience of GPs and PNs during TA epidemics/disaster events/surge events. It is well recognized that the frequency and intensity of disaster events is increasing globally [10]. Early data from TA epidemics pinpoints an increased general practice service demand [6,7,20]. Therefore, it is essential that we understand the experience of general practice staff during these times to improve healthcare delivery [31]. Currently, there are no systematic reviews on the GP response to TA epidemics, with only a single review seeking to understand primary health care and disasters [10].

This novel review does not only aim to link two very different models of care and offer a way forward in dealing with the phenomena of TA, it also aims to outline what we know about primary health care, disaster medical assistance and the gaps in our understanding, as listed in Table 2.

### 4.1. Identification and Treatment of At-Risk Patients

Seasonal increases in pollen counts in the months before a TA event may contribute to the epidemic by priming grass pollen-sensitized patients [9]. Seasonal pollen-initiated asthma often co-exists with allergic rhinitis, and atopic eczema. Several authors have demonstrated how GPs are ideally placed in the community enabling them to identify vulnerable individuals within their practices [33,34,35]. General practitioners can initiate pharmacotherapy or allergen immunotherapy in appropriate patients which has proven efficacy in the control of both allergic rhinitis and allergic asthma [36]. Identification, treatment and education of at-risk individuals (including asthmatics) prior to a TA epidemic unfolding may mitigate the surge of patients presenting to health services.

### 4.2. General Practice Resources

Preparedness (resources and training) for public health emergencies becomes all the more pertinent. This is an area that needs further exploration in the general practice setting, as a lack of preparedness is often cited by GPs as an important factor impacting their response. Studies that questioned GPs about lack of preparedness for respiratory surge events all examined pandemic influenza [26,27]. Thunderstorm asthma epidemics by their very nature are short lived (peak presentations in less than 12 h) yet can produce overwhelming numbers of patients. The only study to examine resource preparedness during a thunderstorm asthma epidemic did so in a hospital setting. The authors clearly demonstrated limitations and challenges with resource management [8]. Caution must be applied when drawing interpretations from respiratory pandemics in which the presentations peak over weeks or months. Hogg et al. surmise that, preparedness for public health emergencies can be underestimated by general practice doctors when it in reality the preparedness (resources and training) of their clinics to respond to disasters is often adequate [26]. On the issue regarding general practice services and disaster preparedness (perception vs reality) there is no clear consensus in the limited research available. To be able to respond to thunderstorm asthma epidemics it is vital to understand if GPs have enough personnel, equipment, space and time. Furthermore, not enough is known as to what experience and training facilitates adequate preparedness for this type of disaster.

### 4.3. GP Education and Training

The knowledge and skills of GPs and their staff are sufficient to manage acute asthma. However, awareness of the phenomena of TA may not be widely known in primary care settings. GP administrative bodies have developed an education and preparedness document to help ready GP clinics and their staff for pandemic influenza [37]. A similar toolkit can be developed for TA. Prior to the environmental pollen season commencing annually, GPs can prepare their clinics with adequate medication and equipment, update clinic procedures (including policies for calling in additional staff after hours and managing patients during surge conditions) and improve their links to emergency departments, ambulance services and community pharmacies.

### 4.4. Integration of GP Services into the Wider Emergency Services Response

In the response and recovery phase, GPs and PNs universally express desire to assist during surge events. There is consensus in the literature regarding this “willingness to help” [22,35,38]. However, very little is known about the facilitators and barriers to the general practice emergency response. Barriers to providing care include, vailability of free healthcare, staff illness, availability of general practice appointments and integration of GPs with wider emergency services.

A minority of GPs hold a Major Incident Medical Management (MIMM) certificate or equivalent and some GPs are part of Australian Medical Assistance Teams (AUSMAT), which are teams available for deployment to international emergency situations. However, for the majority of GPs their role is less clear during public health emergencies [28]. GP organizational structures are not linked into the existing medical response, resulting in GPs being uncertain of their exact role in a mass casualty response. A paucity of adequate operational information may result in reluctance to become involved in a disaster response [5]. The Australian Medical Association recommends that all jurisdictions (Primary Health Networks) maintain a database of GPs who are willing to assist during disasters in their clinics to allow patients to access general practitioners in a crisis [28]. No comprehensive database exists at the time of publication.

### 4.5. Communication

Enhancing surge capacity through communication and co-ordination with emergency services remains an underexplored area. Currently, Australian GPs have access to several resources and online tools to assist their practices with disaster planning, response and recovery, produced by the Royal Australian College of General Practice (RACGP) [37,39,40]. Currently, GP practices develop their own disaster response plans. In some jurisdictions, Primary Health Networks provide support via data collection, identifying gaps and vulnerabilities in the system post-disaster. In Australia, the primary responsibility for managing and coordinating emergency responses lies with multiple state and territory governments and an extensive array of stake holders including State Emergency Services (earthquake, flood, storm and bushfire), Department of Health and Human Services and local health departments in each state(human disease, heatwave) and Country Fire Authority (bushfire). Co-ordinated management between emergency organisations, various levels of government and GP stakeholders remains challenging. While individual patients can be referred to hospital emergency departments by general practitioners during disasters, no direct operational information on unfolding mass casualty events can be dissipated to GPs from the various stakeholders. In other regions, interaction between GPs and emergency service varies greatly. During cyclone Katrina, US family doctors utilized a pre-established hotline to regional emergency services [25]. Unfortunately, Australian and Canadian GPs still feel that gaps in communication remain [26,27].

No effective means has been mentioned in the literature whereby GPs can have two-way communication with medical emergency response services as a disaster is unfolding. Only through investigating the general practice experience during a rapidly unfolding, short-lived and intense disaster, can policy makers conceptualize the most effective means of communication and service co-ordination to help manage the patient surge during these events.

### 4.6. Advocacy, Feedback and Research

A small number of studies looked at the ability of GP clinics to increase surge capacity using adaptive management such as triage, altered procedures/processes, rationing and altering practice hours and shifts. The impact that adaptive management can have on the general practice service response to a TA epidemic is not known.

The majority of literature published in this area are reports from an individual perspective, of a doctor working in a crisis, often outside the clinic environment. Many articles were excluded as mixed samples (GP data included with hospital staff in a hospital setting) added no insights into the challenges of the GP environment. Grey literature similarly often acknowledged the significance of the GP contribution but failed to collect any data or provide a way forward. The published research lacked articles expressing the combined viewpoints of different types of primary health care providers, policy makers, health planners, government and educators, the lessons learned, links to policy, legislation and education [10].

### 4.7. Limitations

The main weakness in a review such as this is in the complexity of incorporating diverse methodologies. While every effort was made to keep the study question focused, combining diverse literature can introduce bias, inaccuracy and can contribute to lack of rigor [19].

The review confirms there are no qualitative experiences on this topic and certainly only a few association studies investigating the general practice data. Closer inspection of the articles suitable for the review, relating to public health emergencies generally, show that three of the studies were found to be of low validity upon appraisal. Additionally, a portion of our conclusions were drawn from grey literature. While the material sourced from grey literature is robust, it often gives an indication of an inferred experience rather than the lived experience of clinicians, as it is not primarily collected for the purpose of research.

Research into disasters, is by its very nature focused on single events and individual case studies, leading to the production of rich data providing details about a moment in time in a unique local context. The studies and government reviews included here, while insightful and interesting, can provide data of limited generalizability not only to other event types but also locations [22,23,24,25]. While every effort was made to select studies, which gave meaningful information about general practice experiences, it may be possible there was some selection bias. It may also be possible that some studies were missed in the search process. A search of this type invariably will find published material which may be subject to a publication bias. In addition, research from non-English sources are missing. Non-English language studies may have provided useful insights as many of these countries may have a greater and well-defined role for general practitioners in disaster management.

## 5. Conclusions

In summary, GPs have a major role to play in the management of patients during a thunderstorm asthma epidemic, as invariably a surge in asthma cases occurs; yet their role in disasters remains poorly defined. General practice is the primary point of access to medical care for many patients during disasters and surge events. Staff have developed a variety of approaches to effectively manage them. Their diversity of skills and knowledge are invaluable assets given the inherent uncertainties of a disaster. Emergency planners should not assume that most victims of a disaster require hospitalization or even emergency department evaluation. Triage and treatment can be effectively undertaken at general practice clinics to avoid overwhelming hospital facilities when large numbers of patients seek medical care at once, yet little is known how to adequately integrate the general practice response into the overall emergency healthcare response. Future studies should focus on gaps identified through this review, particularly the collection of survey data from general practice during surge events and the integration of general practice into the overall emergency healthcare response.

## Figures and Tables

**Table 1:** Characteristics of included studies.

First Author & [Ref No.]	Pub. Year	Type	Disaster Type	Study/Enquiry Subjects	Themes
The Royal Australian College of GP: multiple authors [1]	2004	Report, (grey literature)	Floods, storms, cyclones, bushfires, heatwaves	General practitioners, Australia	There is always an increased use of primary healthcare during a crisis. The role of general practice in the healthcare system during exceptional circumstances is not clearly specified or comprehensively studied. In regional areas, rural GPs usually play a vital role in disaster response. The skills of the rural and remote health workforce (nurses, paramedics and GPs) must be developed and maintained. Additional staffing for GP clinics is essential during disasters. The absence free medical care can deter or delay low-income rural patients from visiting their GP during an emergency. All jurisdictions need to maintain a data base of GPs willing to assist within their clinics and outside their clinics as required.
Hogg, W., et al. [2]	2006	Quantitative cross-sectional self-administered survey. N = 246	Pandemic influenza, SARS, serious respiratory PH emergencies	Family physicians, Canada	The majority of GPs feel that their clinics do not have adequate preparedness for serious respiratory public health emergencies. The majority of GPs (95%) support response measure including: e-mail, fax notices on public health efforts (95%), for physicians (94%), clinical recommendations (92%), internet bulletin boards, discussion groups (40%). The majority of GPs indicate a willingness to help if contacted on an urgent basis in the event of public health emergency or disaster. There are very few structures available for recruiting GPs into the public health response.
Ranse, J. [3]	2012	Qualitative: semi structured telephone interviews, N = 11	Bushfires, Australia	Primary care nursing staff (general practice nurses) (PNs)	General practice nurses (PNs) are educationally prepared and have adequate clinical experience to assist in bushfire disasters. During the bushfire emergency the PNs’ role involved providing psychosocial support, coordination of patient care and clinic resources and as problem solvers. The role involved minimal clinical care.
Teague, B. [4]	2016	Government Report, Hazelwood mine fire (grey lit.)	Coal mine fire	General Practitioners, Australia	During the coal mine fire, a sustained increased demand for general practice services (GPS) was reported in the region, yet little mention is made of the role of GPS in the report. An increase in respiratory illness was noted in the entire region especially in those patients with chronic conditions. The report acknowledges that some general practice data is missing as the Dept Health and Human Services did not collect it post fire. GPs felt public health messages were important as air quality varied over time. Department of Health and Human Services communicated with general practices via health alerts.
Victorian Bushfires Royal Commission [5]	2009	Govt report; various authors (grey lit.)	Bushfires, Australia	General Practitioners, Australia,	GPs provided medical care, material resources, skills, training, patient advocacy and media liaison during and after the bushfires. There is very little mention of the role of GPS in the report.
Robinson, M. [6]	2003	Qualitative: single case study-reflection	Bushfires, Australia	General Practitioners, Australia,	General practitioners have critical roles in the provision of round the clock general medical services to their communities in times of natural disaster. Divisions of general practice have a pivotal role to play in disaster plans.
Edwards, T.D., et al. [7]	2007	Qualitative narrative account. 27 community health centers	Hurricane	Family medicine physicians (community healthcare centers), USA	Primary care physicians are rarely mentioned in medical disaster plans Community (family physicians) effectively cared for the medical needs of disaster victims and prevented emergency departments and hospitals from being overwhelmed. Almost no literature on the role of family physicians in caring for disaster victims. Family physicians used non-traditional triage and treatment spaces in their clinics. The breadth of knowledge and skills that family physicians possess is an invaluable asset given the inherent uncertainties of a disaster.
Pitts, J., et al. [8]	2009	Qualitative, Semi-structured interview, N = 10	Pandemic influenza	General practice registrars, UK	GP registrars identified the following issues and consequences of an influenza pandemic using a simulated disaster planning approach: Stop unnecessary procedures and processes and expect a shortage of medical supplies. Triage, rationing, telephone advice facilities are an effective means to control the patient surge. Clinics could develop treatment criteria as the event unfolds. Doctors should prepare for 50% staff illness and for the consequences of school and childcare closure on staffing. Practice organisation can be altered including hours and shifts.
Dearinger, A.T., et al. [9]	2011	Quantitative, cross-sectional survey, N = 81	Pandemic influenza	Family physicians, USA	Information dissemination efforts between local health departments and primary health care professionals during respiratory surge events are inadequate (as perceived by family physicians [GPs].
Shaw, Kelly A. [10]	2006	qualitative study, semi-structured interview, N = 60	Pandemic influenza	General Practitioners, Australia,	Universal willingness of GPs to provide professional services in a pandemic. GPs are enthusiastic about receiving further information and training in pandemic preparedness. The role of the GPs in responding to pandemic influenza poorly defined. Significant practice preparedness issues were identified by GPs.
Johal, S., [11]	2014	Qualitative, semi-structured interview, N = 8	Earthquake	General Practitioners, New Zealand	All GPs reported a significant increase in workload after the earthquake. GPs often found themselves working outside their area of accustomed expertise. GPs identified a number of coping behaviors. GPs had a greater awareness of self-care strategies that helped them with the workload. The process for identifying hotspots of increased patient demand was poorly coordinated for GPs.
Dept. of Health and Ageing, [12]	2011	Government Review. (grey lit.)	Pandemic influenza	General Practitioners, Australia,	There are very few structures in place to liaise with, support and provide information to GPs during an influenza pandemic. General practice had a larger role than had been considered in the PH planning stage. The Australian General Practice Network (AGPN), and the Royal Australian College of General Practitioners (RACGP), could deliver education, training and reminders to general practice staff.
Davidson, A.C., et al. [13]	1996	Quantitative Questionnaire	Thunderstorm Asthma	ED staff/patients in London hospitals, UK.	Asthma presentations to the ED increased by ten times the expected number from the onset of the thunderstorm. Most respiratory patients could be safely discharged home after an initial course of treatment in ED. Increased presentations were sustained for at least 12 h and up to 24 h post thunderstorm. Demands on ED resources increased in terms of personnel, equipment, medications and space.
Hajat, S., et al. [14]	1997	Quantitative	Thunderstorm Asthma	General Practitioners, UK.	GPs experienced six times the expected increase in asthma presentation the day after a TA epidemic. Consultations on the day of the storm did not increase as the onset was between 6 p.m.–9 p.m. when most practices were closed.
Higham, J., et al. [15]	1997	Quantitative	Thunderstorm Asthma	General Practitioners, deputizing service, UK	A general practice deputising service reported an increase in TA presentations (1500 additional asthma cases in 12 h) The geographical area with increased asthma cases was quite large.
Andrew, E., et al. [16]	2017	Quantitative	Thunderstorm Asthma	Ambulance service data, Australia	Time critical referrals from general practitioners utilizing ambulance services increased by 47% (21% to 80%) during the TA epidemic.

**Table 2:** Gaps identified and future directions.

Disaster Stage	Gaps Identified	Future Directions
Mitigation	Identification of vulnerable groups	1. Improved education and awareness delivered to asthmatic patients about TA by GPs. 2. Clinical guidelines regarding the identification and treatment of allergic rhinitis and atopic patients by GPs.
Mitigation	Identification of high-risk periods for TA	3. Emergency warnings to alert healthcare staff and patients to high pollen days. 4. Improved detection of environmental/weather conditions that may precipitate TA epidemics providing early warning for GPs.
Preparedness	GP and PN education and training	5. Development of TA specific toolkits and factsheets for the recognition, diagnosis and treatment of TA patients. 6. Local knowledge of other emergency services including pharmacy, ED and ambulance access.
Response	Service provision	7. Training programs to enhance clinic surge capacity 8. Understanding of GP team roles and responsibilities during disaster response differ from the normal. 9. Personal preparedness of clinic staff
Response	Service utilization and access	10. Dynamic data base of GP clinics able to treat patients during surge events available to the public.
Response	Resources	11. Guidelines for the adequate management of personnel, medication and equipment in the clinic environment during surge events.
Response	Communication	12. Legislative change to bring GP organizations into a communication loop with emergency service stakeholders similar to CAS (in the UK) but with the potential to provide and receive real time data and feedback from GPs [32]. 13. Communication protocols and guidelines to ensure GPs are provided with the latest operational information as disasters unfold.
Recovery	Advocacy	14. Advocacy by GP organizations for policy and legislative change to better integrate them with emergency service stakeholders and tertiary healthcare providers.
Recovery	Feedback and research	15. Focus on holistic research aiming to understand the larger perspective of TA management (healthcare system capacity and utilization) 16. GP specific research for future planning

## Supplementary Materials

The following are available online at https://www.mdpi.com/1660-4601/17/11/3796/s1, Figure S1: Database search strategy: Ovid MEDLINE. Table S1: Data analysis and themes. Table S2: Quality Assessment of qualitative, quantitative and mixed method studies. Table S3: Quality Assessment for grey literature.
