# Supplementary material for: Thunderstorm Asthma Epidemic—A Systematic Review of the General Practice Perspective
Source: Int J Environ Res Public Health. 2020 May 27;17(11):3796. doi: 10.3390/ijerph17113796 (PMC7312025; doi:10.3390/ijerph17113796)
Supplement: Supplementary file 1 [file ijerph-17-03796-s001.pdf]

Review

Thunderstorm Asthma Epidemic—A Systematic Review of the General Practice Perspective

Ambereen Farouque [1], Rae Walker [1] and Bircan Erbas [1,2,*]

[1] Department of Public Health, School of Psychology and Public Health, La Trobe University, Bundoora, VIC 3086, Australia; amber.farouque@gmail.com (A.F.); r.walker@latrobe.edu.au (R.W.)
[2] Faculty of Public Health, Universitas Airlangga, Surabaya 60115, Indonesia
* Correspondence: B.Erbas@latrobe.edu.au; Tel.: +61-39-479-5657 or +61-39-479-1783

Received: 30 March 2020; Accepted: 25 May 2020; Published: 27 May 2020

Online Supplementary Figure 1.S: Database(s): Ovid MEDLINE(R) ALL 1946 to November 27, 2019
Search Strategy:

#	Searches	Results
1	thunderstorm asthma.mp.	57
2	General Practice/ or Family Practice/	74072
3	1 and 2	1
4	(Primary Health Care or Family Practice or Nurse Practitioner).mp. [mp=title, abstract, original title, name of substance word, subject heading word, floating sub-heading word, keyword heading word, organism supplementary concept word, protocol supplementary concept word, rare disease supplementary concept word, unique identifier, synonyms]	154338
5	1 and 4	0
6	hospital resource management.mp.	5
7	1 and 6	0
8	disaster management, public health emergency, public health disaster, disaster.mp.	0
9	2 and 4 and 8	0
10	2 and 4	66218
11	pandemic influenza, respiratory pandemics,.mp. or Pandemics/	4786

12	10 and 11	5
13	forest fire, industrial fires,.mp.	0
14	heatwaves.mp.	312
15	10 and 14	0
16	hurricane.mp. or Cyclonic Storms/	3808
17	10 and 16	6
18	Floods/ or flooding.mp.	7589
19	10 and 18	1

Online Supplementary Table S.1: Data analysis and themes

**Themes**	**Sub-theme**	**Possible solution/outcome/experience**
Limited resources	Equipment	Rationing equipment<br>Recycling equipment
	Personnel	Stop unnecessary procedures and processes<br>Additional staffing<br>Altered staff roles<br>Triage<br>Developing criteria for treatment
	Space	Use of non-traditional triage spaces<br>Multiple patients to a single treatment room<br>Telephone advice facilities<br>Majority of patients can safely be sent home after a course of treatment.
	Time	Altered working hours and shifts
Increased use of GP services in a public health emergency	Increase in GP consultations during TA epidemic	Role of primary healthcare system during exceptional circumstances is not clearly defined or comprehensively studied<br>Almost no literature on the role of general practitioners in caring for disaster victims.<br>Lack of bulk billing (free treatment) can deter people from visiting their GP during an emergency.
	Increase in after-hours GP consultations observed during TA epidemic	Increased demand on general practice after-hours services as regular practices were closed
Willingness of GP's to provide care	Barriers to providing care	Staff illness.<br>Consequences of school and childcare closure on staffing.<br>Preparedness of general practice clinics perceived as inadequate by GP's
	Facilitators to providing care	GPs uniquely placed to identify vulnerable patients.<br>Willingness of general practice services to provide care<br>Majority of patients could safely be sent home after a course of treatment and majority of asthma management is within the skill set of general practice staff.

	GPs and PNs have the breadth of knowledge, skills and established community/patient links.	GPs and PNs can provide emotional care/patient education/ provide long term care to communities after disaster<br>Nursing roles including a psychosocial supporter, a coordinator of care and resources, and problem solvers
Communication	Facilitators that enhance surge capacity during emergencies	Majority of GPs support response measure: e-mail, fax notices on public health efforts and a hot-line for physicians, clinical recommendations, internet bulletin boards and discussion groups.<br>GPs enthusiastic about receiving further information and training in pandemic preparedness.<br>Public health messages important<br>Agreed communication platforms, channels and protocols exist and are understood and used during emergencies<br>Improve instruction about the event resulting in greater preparedness and a more coordinated response<br>Divisions of general practice have a pivotal role to play in disaster plans
	Barriers to increasing surge capacity	GP perceive information dissemination efforts from local health departments during respiratory surge events are deficient<br>Very little public health structures in place for GP or PN recruitment into public health emergency response teams.
Co-ordination	Facilitators that enhance surge capacity during emergencies	Appropriate personnel are involved and informed during emergencies.<br>Planning between Department of Health and Human Services and general practices, to ensure a better utilisation of medical expertise.
	Barriers to increasing surge capacity	Very little mention of the role of general practice services.<br>General practitioner services are rarely mentioned in medical disaster plans

Online Supplementary Table S.2.: Grey literature appraisal- Public Health Ontario Guide to appraising grey literature

Title & [Ref. no.]	Author/Publisher Validity Questions: Is the information shared for commercial purposes? Does the site sponsor have a political or philosophical agenda?	Host/Sponsor Validity Questions: Does the author/host have an agenda? Publishing body: Are they a well-known provincial/national government-affiliated public health agency, for-profit company. Political or philosophical agenda?	Facts/References Validity Questions: Are references to research evidence? Is the information based on research/data/analysis or is it opinion piece? Can you verify some cited sources, facts? How current are the references?	Currency Validity Questions: Publication date? Is the information current?	Overall Validity
Hazelwood Mine Fire enquiry [4]	Victorian Government autonomous body with independent authorship panel, expert evidence, testimonies and independent investigators.	Victorian Government, agenda is to produce an independent National Government enquiry into the Hazelwood Mine Fire.	References all based on direct investigation, analysis or research evidence. All sources can be verified. References current	2016	High
Victorian Bushfires Royal Commission [5]	Victorian Government autonomous body with independent authorship panel, expert evidence, testimonies and independent investigators.	Victorian Government, agenda is to produce an independent National Government enquiry into the Victorian bushfires	References all based on direct investigation, analysis or research evidence. All sources can be verified. References current	2009	High
Rural Communities and Disaster Recovery: Position Paper [1]	National Rural Health Alliance including; The Royal Australian College of General Practitioners: multiple authors, Sponsors support rural communities' health.	Multiple Rural Health Stakeholder Sponsors represent health consumers, health care professionals, and service providers including the RACGP; Political agenda is to lobby government in support of rural health priorities.	References current and some based on stakeholder data. Majority of sources cited or government references	2004	High

Review of Australia's health sector response to pandemic (H1N1) 2009: lessons identified.". [12]	Dept. of Health and Ageing, Canberra, Australia.	National Government Departmental Review: agenda is to produce an account and analysis of the pandemic 2009. Multiple stakeholder consultation	References all based on departmental data sources, direct investigation, analysis or research evidence. Majority of sources cited government references or data. All external sources can be verified and current	2011	High

Online Supplementary Table S.3: Quantitative, qualitative and mixed methods article appraisal - Public Health Ontario MetaQAT (quality appraisal tool)

Ref ID	Publication Year	Citation	Validity
[2]	2006	Hogg, W., et al. (2006). "Enhancing public health response to respiratory epidemics: are family physicians ready and willing to help?" Canadian Family Physician 52(10): 1254-1260.	Overall appraisal 13/13, High
[14]	1999	Hajat, S., et al. "Association of air pollution with daily GP consultations for asthma and other lower respiratory conditions in London." Thorax 54(7): 597-605.	Overall appraisal 12/13, High
[15]	1997	Higham, J., et al. "Asthma and thunderstorms: Description of an epidemic in general practice in Britain using data from a doctor's deputising service in the UK." Journal of Epidemiology and Community Health 51(3): 233-238.	Overall appraisal is 12/13, High
[13]	1996	Davidson, A. C., et al. "A major outbreak of asthma associated with a thunderstorm: experience of accident and emergency departments and patients' characteristics. (Thames Regions Accident and Emergency Trainees Association)." British Medical Journal 312(7031): 601.	Overall appraisal is 11/13, High
[3]	2012	Ranse, J., & Lenson, S. (2012). Beyond a clinical role: nurses were psychosocial supporters, coordinators and problem solvers in the Black Saturday and Victorian bushfires in 2009. Australasian emergency nursing journal, 15(3), 156-163.	Overall appraisal is 7/13, Low
[6]	2003	Robinson, M. (2003). Bushfires, 2003: a rural GP's perspective. Australian Family Physician, 32(12), 985-988.	Overall appraisal is 7/13, Low
[7]	2007	Edwards, T. D., Young, R. A., & Lowe, A. F. (2007). Caring for a surge of Hurricane Katrina evacuees in primary care clinics. The Annals of Family Medicine, 5(2), 170-174.	Overall appraisal is 7/13, Low
[8]	2009	Pitts, J., Lynch, M., Mulholland, M., Curtis, A., Simpson, J., & Meacham, J. (2009). Disaster planning: using an 'evolving scenario' approach for pandemic influenza with primary care doctors in training. Education for Primary Care, 20(5), 346-352.	Overall appraisal is 10/13, High
[9]	2009	Dearinger, A. T., Howard, A., Ingram, R., Wilding, S., Scutchfield, D., Pearce, K. A., & Hall, B. (2011). Communication efforts among local health departments and health care professionals during the 2009 H1N1 outbreak. Journal of Public Health Management and Practice, 17(1), 45-51.	Overall appraisal is 9/13, High

[10]	2006	Shaw, K. A., Chilcott, A., Hansen, E., & Winzenberg, T. (2006). The GP's response to pandemic influenza: a qualitative study. Family Practice, 23(3), 267-272.	Overall appraisal is 12/13, High
[11]	2014	Johal, S., Mounsey, Z., Tuohy, R., & Johnston, D. (2014). Coping with disaster: General practitioners' perspectives on the impact of the Canterbury earthquakes. PLoS currents, 6.	Overall appraisal is 11/13, High
[16]	2017	Andrew, E., Nehme, Z., Bernard, S., Abramson, M. J., Newbigin, E., Piper, B., ... & Smith, K. (2017). Stormy weather: a retrospective analysis of demand for emergency medical services during epidemic thunderstorm asthma. *bmj*, *359*, j5636.	Overall appraisal is 9/13, High
